# Draft Genome Sequence of the Predatory Marine Bacterium *Halobacteriovorax* sp. Strain JY17

**DOI:** 10.1128/genomeA.01416-17

**Published:** 2018-01-04

**Authors:** Jonathan M. Young, Timofey Skvortsov, Ksenia Arkhipova, Christopher C. R. Allen

**Affiliations:** a School of the Biological Sciences, Queen’s University Belfast, Belfast, United Kingdom

## Abstract

A draft genome sequence of *Halobacteriovorax* sp. strain JY17 was assembled from a metagenomic data set. The 3.47-Mbp genome of this unusual predatory bacterium contains 3,263 protein-coding sequences, 33 tRNAs, and 2 copies each of the 16S, 23S, and 5S rRNA genes. This is only the third sequenced representative of this genus.

## GENOME ANNOUNCEMENT

The genus *Halobacteriovorax* comprises predatory deltaproteobacteria found in marine environments (1). They prey on Gram-negative bacteria, penetrating the cell wall and multiplying within the periplasmic space (2, 3), and have been identified as potential biocontrol agents (4). Here, we present *Halobacteriovorax* sp. strain JY17, assembled from a methanol-enriched seawater sample from the Irish Sea. To our knowledge, this is only the third available genome for a member of the genus *Halobacteriovorax* to date.

Methanol enrichment cultures were set up according to a previously described method (5) and incubated in a shaking incubator for 18 days at 22°C and 50 rpm, after which DNA was extracted from cell pellets using the PowerSoil DNA isolation kit (MO BIO Laboratories, Carlsbad, CA, USA). Metagenomic DNA libraries were prepared using an Illumina Nextera DNA library kit and sequenced on an Illumina NextSeq 500 DNA sequencer (Illumina, San Diego, CA, USA) in mid-output mode using a paired-end flow cell (2 × 150 bp read length, V2 chemistry) at the DNA Sequencing Facility, Department of Biochemistry, University of Cambridge (Cambridge, UK).

Raw reads from three individual samples were pre-processed using BBTools (6) and coassembled using metaSPAdes (7). Contigs were taxonomically assigned using Kaiju (8) and binned on the basis of GC% and differential coverage using CONCOCT (9).

The resulting bins were manually refined to reduce contamination using Anvi’o (10); briefly, for each bin, contigs were clustered on the basis of GC% by Euclidean distance and Ward’s linkage clustering followed by visualization of the resulting dendrograms and manual removal of any outliers. Reads mapping to contigs within each genome bin were extracted and reassembled using SPAdes (11) with the “-careful” flag enabled. A final scaffolding step was performed using SSPACE (12). Final draft genome completeness and contamination was assessed using CheckM (13). The final draft genome was annotated using the NCBI Prokaryotic Genome Annotation Pipeline (PGAP) (14) and reviewed using RAST v2.0 (15).

This resulted in a 3.47-Mbp draft genome estimated to be 93.9% complete with 1.79% potential contamination, comprising 5 scaffolds with a GC content of 36%, 3,354 coding sequences, 34 tRNAs, and 2 copies of 16S, 23S, and 5S rRNA genes. Interestingly, only 26% of the coding sequences could be assigned to SEED subsystems, indicating an unusually high level of novel functional diversity.

BLAST alignment of the 16S rRNA genes indicated 98.7% similarity to *Halobacteriovorax marinus*; however, further investigation with gANI (16) revealed a 77.6% average nucleotide identity and an alignment fraction of 0.76 between the two genomes. Additionally, species assignment using specI (17) revealed an average identity of 86.5% to *H. marinus* across 40 conserved housekeeping genes. Therefore, we propose that the newly obtained genome represents a new species within the genus *Halobacteriovorax*. Furthermore, our data indicate that alignment of the 16S rRNA gene may be a poor method of delineating species level diversity in the genus *Halobacteriovorax*.

### Accession number(s).

The metagenome-derived draft genome sequence of *Halobacteriovorax* sp. strain JY17 has been deposited in DDBJ/EMBL/GenBank under the accession number NJER00000000.
